# Collectanea: Miscellaneous

**Published:** 1835-04-01

**Authors:** 


### MISCELLANEOUS.

### VARIETIES OF RHUBARB.

According to M. Geiger, ioduretted hydriodic acid is a good re-agent to distinguish the different rhubarbs of commerce; it gives a green tint with the decoction of Russian rhubarb; a brownish one with the Chinese; a deep red with the English, and a blue with the French (Jour. de Chimie Méd., vi. 535.) The same author is of opinion, that iodine will show whether rhubarb will keep long or not, as this depends on the great or less quantity of fecula contained in it (*ibid.*) which renders it more or less susceptible of being punctured by the insect called *Sinodendrum pusillum*, Kirby. Thomson assures us, that a solution of isinglass caused a greater precipitate in the infusion of Chinese than of Turkey rhubarb, and that the decoction of yellow bark throws down an abundant greenish precipitate in Russian rhubarb: in the Chinese it is of a brilliant yellow.

[From the Dictionnaire Universel de Matière Médicale, par F. V. Mérat et A. J. de Lens; tome vi. p. 65. This useful work, of which we gave a notice in our first Number, is now completed, in six volumes.—Edit. *Med. Quart. Rev.*]

### PRESERVATION OF WOOD BY CORROSIVE SUBLIMATE.

After immersing the wood in a solution of the corrosive sublimate, it still remains in a state to influence chemical tests or agents; so that at any time it will be easy to detect, by a simple experiment, whether timber has been prepared by Mr. Kyan's process. The effect may be observed by taking a piece of simple deal, that has not undergone the process, and applying a few drops of hydro-sulphuret of ammonia, when no effect will be produced; but when the same test is applied to a piece of wood which has been immersed in the solution, a dark stain will be instantly left upon it. Thus the presence of the protecting substance is proved by the contact of a little hydro-sulphuret of ammonia, occasioning no change in the simple wood, and producing a black mark instantaneously on its application to wood previously immersed in the solution of corrosive sublimate. This evidently shows that among the fibres in the very substance of the wood, some of the calomel remains behind. It was the case with some specimens of linen examined by Professor Faraday, which had been long immersed in the solution of corrosive sublimate. Upon simply washing in water, he found they yielded no evidence of any process having taken place, but when he applied nitric acid he obtained an action of the mercurial matter within the substance, and rendered it evident.—*Dr. Birkbeck's Lecture on the Preservation of Timber, by Kyan's Patent.*

[This is a remarkably interesting pamphlet.—Ed. *M. Q. Rev.*]

### CHEMICAL EFFECTS OF ELECTRICITY.

Under the head chemical effects, we may perhaps speak of the increased rapidity with which putrefaction proceeds in all animal or vegetable bodies which have been killed by electricity. It is also well known that a storm frequently gives a putrid smell to meat; and grain under fermentation, as in the process of brewing, suffers a very sudden change, and is often rendered useless. M. Achard, of Berlin, recently investigated this subject with a view to determine the influence of electricity, which is always present in the atmosphere in stormy weather, in producing these effects. By his experiments, he was led to the conclusion that whenever flesh is electrified, or animals killed by electricity, the progress of corruption is greatly accelerated, and that the putrefaction of flesh after a storm must be ascribed solely to the more abundant accumulation of the electric matter at that time.—*Higgins' Alphabet of Electricity.*

### CLIMATE OF MADEIRA.

Dr. Renton's report of the effects of the climate of Madeira on the invalids who passed the last winter there has just reached us. The total number of pulmonary invalids who arrived there during the season of 1833-34 was sixty-six. Of this number fifteen died; forty-three returned to their homes; and eight still remain in the island. "Of the fifteen fatal cases," says Dr. Renton, "thirteen ought not to have left their homes; of the forty-three who left the island for England, or other parts of the world, thirty-six were very much improved; indeed, I may say, a large majority of them went away well." The result was very different a few years since, when persons were only sent to Madeira in the advanced stage of the disease.—*Dr. Clarke in Cyclop. of Pract. Med.* part xxiii.

MEDICO-LEGAL DISINTERMENTS.

I shall give a few examples of what may be effected by researches of this kind. Not long ago, proofs of poisoning were discovered in the remains of a body that had lain in the earth for seven years. The case occurred at Lyons. A woman had long been suspected of having poisoned her father, but, for want of direct proof, justice could not reach her. At length a circumstance occurred which threw a strong light upon her guilt; and, though seven years had elapsed, it was thought requisite and proper to disinter the body. The examination and the necessary experiments were conducted by M. Ozanam, assisted by M. Ide; and complete evidence of the presence of arsenic among the shapeless tissues of the stomach was obtained.

There was a very singular case investigated in Paris during the last year. I allude to that in which the remains of a murdered female were disinterred, identified, and the mode of death ascertained, after a lapse of eleven years. Little more than a heap of bones was found; but, from their position, condition, and several peculiarities, the reporters (among whom we find the names of Orfila, Barruel, Chevallier, and Boys de Loury,) were enabled to come to the following conclusions: 1. That those were the bones of a human skeleton; 2. of a female; 3. the age from sixty to seventy; 4. the stature about five English feet; 5. the hair had been bright blond, but latterly was mixed with grey; 6. the deceased had died of strangulation, the act being homicidal; and, 7. the remains had lain in the earth for several years.

The prisoners, who had long been suspected of the murder, were tried, and condemned to the gallies for life, having had a narrow escape of the guillotine.

It may be perhaps curious, and not uninteresting to you, to be reminded of certain disinterments which have taken place in this country for medico-legal purposes: we have some very remarkable ones on record. As a counterpart, and a sort of contrast to the 1st case, let me call your attention to what occurred in the year 1758, at Knaresborough, in Yorkshire. I allude to the celebrated case of Eugene Aram, around whose history the pen of the novelist has recently thrown so much attraction, but whose plain unvarnished story is in itself, independent of any collateral fiction, almost a romance. The highly-gifted but unfortunate person, instigated by penury and jealousy, as it is said, but, above all, by the instigation of a depraved and profligate companion, committed a murder on one Daniel Clarke; and it was not till fourteen years after that proof of the crime was procured: that proof was the skeleton of the victim. Suspicion had never been attached to the name of Aram, but the desperate Houseman was long supposed to have been the assassin. At length, a skeleton being found accidentally in a field at Thistlehill, near Knaresborough, it was thought to be Clarke's, and Houseman was examined at the inquest. He was much confused and conscience-stricken on the occasion; but, affecting a degree of levity which had just the contrary effect to what he intended, he took up one of the bones, and said, "This is no more Clarke's bone than it is my bone." Upon which he was immediately questioned as to where Clarke's bones were, for he had now as good as confessed that he knew. He turned king's evidence, denounced Aram as the murderer, admitted that he had been an accomplice, and described exactly where the true skeleton would be found. In St. Robert's Cave, he said, they would find it, "just by the entrance, and with the head turned towards the right."

On the trial the skull was produced in court,—a ghastly witness against the accused. On the left side of it there was a fracture, that, from its nature, could not have been made but by the stroke of some blunt instrument; the piece was beaten inwards, and could not be replaced but from within. Mr. Locock, the surgeon, who was the chief, if not the sole, medical witness examined as to the appearances, give it as his opinion, "that no such breach as that pointed out in the skull could have proceeded from natural decay,—that it was not a recent fracture by the instrument with which it was dug up, but seemed to be of many years' standing." This seems to have been about the amount of the evidence, which, taken with the circumstantial detail given by Houseman and some other witnesses, operated in procuring the prisoner's conviction: if, indeed, Aram did not himself do more to that end, by the extraordinary and truly surprising defence which he made, at once singular for its eloquence and its learning. This, I may remark, was the opinion of the celebrated Dr. Paley, who happened to be present at the trial. Many years afterwards when conversing with some friends, about certain lives in the Biographia Britannica, which somebody observed were those of obscure individuals, Eugene Aram's, for instance. "Nay," said Paley, "a man that has been hanged has some pretensions to notoriety, and especially a man who has got himself hanged by his own cleverness, which Eugene Aram certainly did."

Aram called no witness for the defence. He relied wholly on his own ingenuity, and produced, certainly, most powerful arguments to shew that the bones discovered were those of some hermit, who had in former times dwelt in the cave; and he mentioned several cases similar to St. Robert's, in which human bones had been found. When we recollect that it is the great principle of our law, that no man can be condemned for murder unless the body of the person supposed to have been murdered be found and identified, we must at once admit the importance of the line of argument taken up by the accused.

The identity, in fact, of the skeleton was not made out: so far from its being proved to be Clarke's, it was not even shewn to be that of a man; the sex was not proved, nor the age, nor, in short, any of the numerous points which, no doubt, would be elicited, were the examination instituted at the present day. All that was ascertained was, that here was a human skeleton, found as directed to be sought for by a wretch who had given information, in expectation of pardon for his crimes. Why might it not be wholly a plot of Houseman's, to get rid of the unfortunate Aram? He might have buried the bones there himself, or found them buried when digging in the cave, perhaps, for the concealment of plunder.

Let it not be thought that I am of the number of those who hold Aram to have been guiltless. The man is said to have confessed his crime before his execution. But my object in dwelling on the circumstances is to shew the very insufficient circumstantial testimony on which the prisoner's life was forfeited, under the semblance of law and justice. The truth seems to be (as Paley said, and as now seems generally admitted,) that Aram was chiefly indebted to his own eloquence and cleverness for the forfeiture of his life.

About the close of the seventeenth century occurred the deeply interesting trial of Mr. (afterwards Judge) Cowper, for the murder of a young Quaker lady. There can at present be little doubt but that the deceased drowned herself in a fit of disappointed love: at the trial, however, and for a long time after, much contrariety of opinion prevailed as to the question, whether she was simply drowned, or murdered first, and thrown into the river afterwards. The evidence for and against the prisoner was chiefly derived from the appearances of the body, disinterred six weeks after burial. No water was found in the stomach and lungs; a fact deemed conclusive by one party that the lady could not have been drowned; but competent witnesses on the other side, among whom were Garth, Sir Hans Sloane, and Cowper, the celebrated anatomist, shewed, according to a more correct pathology, that the introduction of water into the cavities on submersion is purely accidental, and that there were appearances enough in this case to indicate suicidal drowning. The lungs, stomach, and abdominal viscera, I may add, were found perfectly sound at the time of the medico-legal examination.

In the year 1828 there was another remarkable case, in which the chief evidence for the prosecution was derived from a medico-legal disinterment. You will probably remember the circumstances of the trial of Corder, for the murder of Maria Martin. The body of the victim was found buried in a barn, where it had lain for eleven months. On the trial, Mr. Lawton, the surgeon, stated, that he first saw the body in the hole where it was discovered. It was much decomposed, and had the appearance of having been buried for nine or ten months at least. He then described the dress, and gave so many particulars relative to what he observed, that the deceased was completely identified, and the manner of her death exactly made out. A handkerchief was found tied tightly round the neck, forming a groove. In the neck there was also the mark of a perpendicular stab, about an inch and a half in breadth, extending deep into the neck. There was an appearance of injury to the right eye, and the right side of the face; it seemed as if something had passed in at the left side, through the cheek, removing the two last grinders, and then out at the right orbit. This was apparently done by a pistol-ball. The lungs and chest were also examined, and there was found a wound between the fifth and sixth ribs, penetrating into the substance of the heart. This was effected by a sword, which was produced.

The sword was identified in court by the cutler who had sharpened it for Corder; and the head of Maria Martin was also identified, chiefly by the loss of a tooth in the upper and lower jaw. The observations made in the lungs, too, were important; there were adhesions of the pleura, and other marks, by which it appeared that the deceased had had cough and pain of the chest not long before death, which was precisely Maria Martin's case.—*Dr. Cummin, in Med. Gazette.*

### CASES OF PERSONS FALSELY IMPRISONED AS LUNATICS.

Pinel is equally in favour of removal, and of asylums, when well conducted; but intimates that the interest of the proprietors is opposed to that of their patients. He says, p. 52, " I was engaged to attend, in a professional capacity, at an asylum, where I made observations upon this disease for five successive years. My opportunities for the application of moral remedies were, however, not numerous. Having no part of the management of the interior police of that institution, I had little or no influence over its servants. The person who was at the head of the establishment had no interest in the cure of his wealthy patients, and he often unequivocally betrayed a desire that every remedy should fail." As human nature is the same every where, there can be no doubt that what is true in France is equally so in England, and that lunatic asylums of every description, and especially private ones, ought to be placed under very strict police superintendance. Certainly it is the interest of the patients to be cured as speedily as possible; whilst it is as evidently the interest of the proprietors to retain their patients, especially their wealthy ones, during the remainder of their days; in which sinister purpose they are very frequently seconded by other interested parties. Whilst writing these pages, the author was attending a lady for a disease of the skin, who, two years previously, had been dismissed from a lunatic asylum by the visiting commissioners of the College of Physicians, London, after having been confined therein five years by her husband, in order that he might live undisturbed in open adultery with another woman. This unfortunate patient is the mother of eight children, and rather weak in her intellects, but in no respect to such a degree as to justify her being confined in a mad-house: on the contrary, she conducted her domestic affairs, and brought up her family with credit, till her husband became fascinated with a lady of superior attractions. He then found out that his wife was a fit inmate for one of those large establishments "in and about the metropolis;" and no doubt he thought it very "fortunate" that their doors stand, like traps, ever ready to open inwards.—*Dr. Gaitskell, on Mental Derangement.*

